# Toxins Secreted by *Bacillus* Isolated from Lung Adenocarcinomas Favor the Penetration of Toxic Substances

**DOI:** 10.3389/fmicb.2015.01301

**Published:** 2015-11-23

**Authors:** Alexandra Merlos, Pau Rodríguez, Iván Bárcena-Uribarri, Mathias Winterhalter, Roland Benz, Teresa Vinuesa, Juan A. Moya, Miguel Viñas

**Affiliations:** ^1^Molecular Microbiology and Antibiotics, Department of Pathology and Experimental Therapeutics, Medical School, University of BarcelonaBarcelona, Spain; ^2^Department of Thoracic Surgery, Hospital Universitari de Bellvitge, University of Barcelona-IDIBELL-HUBBarcelona, Spain; ^3^MoLife, Jacobs UniversityBremen, Germany; ^4^Cooperativa de Ensino Superior Politécnico e Universitário, IINFACTSGandra, Portugal

**Keywords:** cigarette, lung cancer, *Bacillus*, toxin, transmembrane channels

## Abstract

The aim was to explore the eventual role of bacteria in the induction of lung cancer by smoking habits. Viable bacteria closely related to the genus *Bacillus* were detected at high frequencies in lung-cancer biopsies. Similar, if not identical, microbes were isolated from cigarettes and in smog. Bacteria present in cigarettes could be transferred to a physiological solution via a “smoker” device that mimicked their potential transfer during smoking those bacteria produce exotoxins able to open transmembrane pores. These channels can be used as a way to penetrate cells of benzopyrenes and other toxic substances present in tobacco products. We hypothesize that Bacillaceae present in tobacco play a key role in the development of lung cancer.

## Introduction

Lung cancer is heterogeneous, complex and involves alterations at multiple levels (genetic, epigenetic, protein expression; Cooper et al., 2013). Factors influencing the emergency of lung cancer are also heterogeneous (Yang et al., 2013). Among them, smoking habit has been epidemiologically described as the most prominent (Hecht, 2012). Lung cancers develop through a multistep process which involves growth promoting pathways and inhibition of tumor suppressor pathways due to alterations at genetic and epigenetic levels. A better understanding of causes and biochemical pathways involved in lung cancer is needed to improve treatment strategies. Interruption of the downstream biochemical pathways activated by drive tumor growth is one of the main fields of biomedical research. Despite the great progress made in several areas of oncology, the treatment and outcome of lung cancer has not improved significantly except surgical approaches. Lung cancer is a relevant and increasing cause of cancer death. Increase of understanding of the biological pathways involved in lung cancer etiology is required to identify key biomolecules of clinics, but also targets for the development of novel therapies. Undoubtedly, it has been pointed out that smoking is a main cause of lung cancer. Thus, smoking avoidance strategies are being one of the main weapons we have to fight (or to prevent) lung cancer. This disease is, like all tumors, caused by underlying abnormalities in DNA sequence. From a histopathological point of view lung cancers are classified as non-small cell lung cancer (NSCLC) and small cell lung cancer (SCLC). Over the last few years and in conjunction with the noticeable change in the smoking habit, there has been a change in cigarette composition (curing, filtering, etc.). Furthermore, there has also been a shift in the predominant histology pattern (NSCLC). This way, adenocarcinoma has become the most frequent type of NSCLC.

A link between bacterial infection and carcinogenesis has been described most notably for *Helicobacter pylori* and gastric cancer. Similarly, there have been some recent breakthroughs, where molecular pathways involving mycobacteria in carcinogenesis have been identified (Holla et al., 2014), or determining the presence of lipopolysaccharide inhaled through smoke with the development of lung carcinoma in animal model (Melkamu et al., 2013).

Recently it has been pointed out that long-term infection by *P. gingivalis* of oral cancer cells induces an increase in the expression level of CD44 and CD133, well-known cancer stem cell markers, and promotes the tumorigenic properties of infected cancer cells compared to non-infected controls (Ha et al., 2015). Moreover, *P. gingivalis* has been reported also as a noncanonical activator of β-catenin and inductor of disassociation of the β-catenin destruction complex by gingipain-dependent proteolytic processing. β-Catenin activation in epithelial cells by *P. gingivalis* may thus contribute to a proliferative phenotype (Zhou et al., 2015). Other oral bacteria such as *Fusobacterium nucleatum* have been also related with cancer since they stimulate human OSCC (Oral Squamous cell carcinoma) proliferation inducing the expression of key molecules involved in tumorigenesis (Binder Gallimidi et al., 2015). Evidences of *Propionibacterium acnes* infections in human prostate tumors have been also reported (Chen and Wei, 2015).

There is considerable evidence that inhaled toxicants such as cigarette smoke cause changes into the genetic material (mutations) and also changes to the epigenetic landscape (DNA methylation, chromatin state, and eventually others; Hecht, 2012). These alterations lead to the appearance of diseases such as lung cancer and chronic obstructive pulmonary disease (COPD). Both diseases are strongly epidemiologically linked to cigarette smoking. However, the precise mechanisms by which tobacco causes these pulmonary diseases have not been fully established yet. Chemical mechanisms exploration has shown the strong relationship between some toxic substances present in the tobacco and cancer. Among them the best known molecular group is the one comprising benzopyrenes. Benzopyrenes are pentacyclic hydrocarbons comprising both pyrene and phenylene groups they are also called polycyclic aromatic hydrocarbons. Benzopyrenes have been described as agents playing a relevant role in the origin of lung cancer (Denissenko et al., 1996). In this work we have explored a possible mechanism by which smoking can favor the emergence of lung cancer. As it has been demonstrated in some other cancers, here again bacteria seem to play a key role.

## Materials and methods

### Bacteriological media and chemicals

Tryptic soy broth (TSB), Luria Bertani broth (LB), Sabouraud-chloramphenicol, Mueller-Hinton agar (MHA), Ringer's solution, and thioglycollate broth were from Sharlau (Barcelona, Spain). Minimal defined agar medium was prepared (per L) from 20 mL of Vogel-Bonner E Medium × 50 and 50 mL of 10% glucose (w/w). Top molten agar contained (per L) 6 g of NaCl, 0.05 mM histidine, 0.05 nM biotin, and 6 g of agar. Chemicals were from Sigma-Aldrich (St. Lois Mo), and Fluka (Munich, Germany).

### Ames test

Supernatants of all isolates were tested for direct mutagenicity using the following strains: *Salmonella enterica*, Pattee TA98, TA100, TA1535, and TA1537 and *Escherichia coli*, strain 7326 as published previously (Ames et al., 1973; Mortelmans and Zeiger, 2000).

### Bacteria in cigarettes

Cigarettes and rolling tobacco of 25 different brands from nine different tobacco shops in Barcelona were cut in 1 cm length pieces; tobacco was removed and weighed. Equivalent weight of rolling tobacco was measured out, both in sterile environment of a laminar flow hood. Tobacco samples were transferred into tubes containing 5 ml of Ringer ¼. After 5 min soaking, the suspensions were subjected to ultrasonic water bath for 30 s, vortexed for 2 min, and then centrifuged at 3000 rpm for 1 min. The supernatants were collected and spread (100 μl of each) onto three plates containing TSA, or Sabouraud-chloramphenicol agar and into thioglycollate broth. Samples were incubated at 37°C for 24 h (but Sabouraud-cloramphenicol, which was incubated 48 h at 30°C). Colonies on the plates were scored and their macroscopic appearance was recorded. Bacteria were visualized under the microscope after Gram staining. Spore production, type of energy metabolism, biochemical test responses, and 16S RNA sequence were determined.

### Smoker device

**Figure 1:**
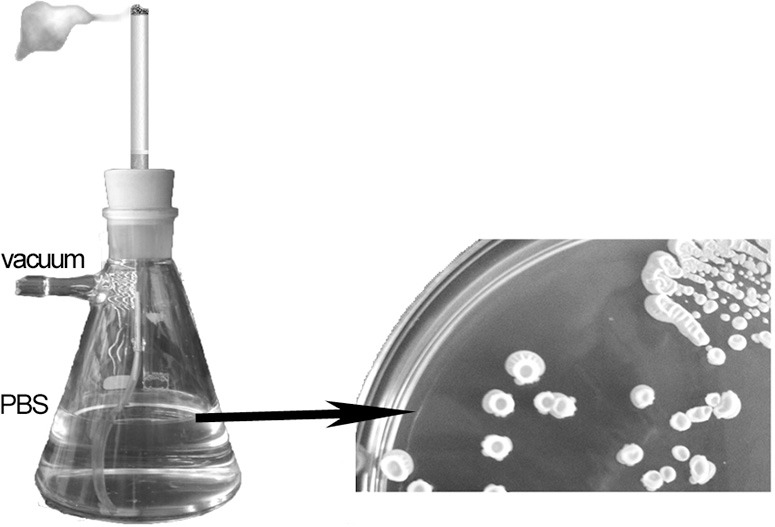
**Smoking device to transfer tobacco products from cigarrette to 100 ml of Phosphate buffer saline**.

A smoker device was constructed using a Kitasato flask containing 100 ml of Phosphate buffer saline (PBS) and connected to a vacuum system. Cigarettes were placed in a holder and artificially “smoked” such that the “inhaled” particles were trapped in the PBS (Figure 1). Aliquots of “smoker PBS” were then inoculated onto the above-described media. Initial experiments showed that relevant data were obtained only from the TSA plates such that in subsequent experiments only these plates were inoculated. Colonies were scored after incubation of the samples at 37°C for 24 h. The identities of the isolates were confirmed by matrix assisted laser desorption ionization time-of-flight (MALDI-TOF).

### Patient selection

Patients undergoing thoracic surgery for tumor exeresis were included, except those with previous history of chemotherapy or radiotherapy; inmunitary-based diseases or HIV infected; respiratory secondary infections during the previous 2 weeks before operation and/or under antibiotic therapy.

### Tumor biopsies

Tumor tissue was obtained from 30 patients who underwent thoracic surgery for the removal of a primary lung tumor at the Hospital Universitari de Bellvitge (HUB). Two tumor samples were obtained from each patient, one for pathological analysis by the hospital pathologist and another for our microbiological study. From the latter, one half was used for polymerase chain reaction (PCR) and other analyses, and the other half for microbiological culture. The surgically removed tumor samples were immediately placed in 5-ml tubes containing 1 ml of Ringer prokaryotes medium and transported as quickly as possible, and always within the first 2 h after the operation, to our laboratory. Tissue for the PCR and other analyses was stored frozen at −80°. Tissue for microbiological culture was cut into thin slices using a surgical knife, transferred in a sterile atmosphere to the above-described solid and liquid media, and incubated as described above. All experiments were performed in accordance with relevant approved guidelines and regulations. Experimental protocols were previously approved by the *Comité Ético de Investigación Clínica (CEIC) del Hospital Universitario de Bellvitge*. Informed consent was obtained from all patients whose biopsies were studied, patients were previously informed.

### Bacteriological characterization

Biofilm formation by the isolates in the different media was determined in at least four replicate wells and in triplicate experiments. The mean absorbance or fluorescence value was corrected by subtracting the absorbance or fluorescence reading of the respective non-inoculated control prior to the statistical analysis. Based on the evaluation criteria of Stepanovic et al. (2000), adherence was classified as: none, weak, moderate, or strong, as evaluated from optical density (OD) measurements. The cut-off point of the OD (ODc) was three standard deviations above the average of the OD of the negative controls.

### Toxin detection and characterization: planar lipid bilayer preparation

The pore-forming activity of the supernatants from cultures was assayed; supernatants were precipitated with polyethylene (PEG) 3350 and dialyzed against water and TRIS-EDTA. The resulting material, referred to as SR, was tested for its pore-forming activity by two different methods: multichannel solvent-containing membranes and single-channel solvent-free membranes. Single channel conductance was measured in both cases. Ion selectivity, voltage-gating, and substrate specificity were determined using multichannel solvent-containing membranes.

### Single-channel conductance

Black lipid bilayers were prepared as described previously (Benz et al., 1978; Benz, 2001). Membranes were formed by spreading a 1% (w/v) solution of 1,2-diphytanoyl-*sn*-glycero-3-phosphocholine (DphPC; Avanti Polar Lipids, Alabaster, AL) in *n*-decane across the aperture to form a lipid film. The aqueous solutions on both sides of the membrane were buffered with 10 mm MES-KOH to pH 6. Toxin was added to the *cis* side. Membrane conductance was measured after the application of a fixed membrane potential using a pair of Ag/AgCl electrodes (with salt bridges) connected in series to a voltage source and a homemade current amplifier. The amplified signal was monitored on a storage oscilloscope (OWON, Chorley, UK) and recorded on a strip chart recorder (Rikadenki, Freiburg, Germany).

Voltage gating of the channels was monitored following a method described elsewhere (Ludwig et al., 1986; Bárcena-Uribarri et al., 2010) using membrane potentials within a range of −100 to +100 mV. Membrane conductance (*G*) as a function of voltage (*Vm*) was measured when the opening and closing of the channels had reached equilibrium after the decay of the membrane current due to the voltage step. The initial conductance (*Go*) was obtained immediately after the onset of the voltage and was linear with respect to the voltage. Gating of the channel by dividing *Go* by the subsequently measured conductance (*G*).

### Pore size determination

Pore size was estimated by recording the multichannel bilayers in bathing solutions containing 20% of a non-electrolyte (NE) with a defined radius and molecular weight. Under these conditions, the conductance responds as a function of the permeability of the NE, which in turn depends on its and on the channel diameter. The latter should be approximately equal to the smallest NE that does not enter the channel and, therefore, does not reduce its conductance. The procedure was described previously (Krasilnikov et al., 1992, 1998). The NE-containing bathing solution was 1 M KCl to which 20% of the NE was added. The size of a possible constriction inside the channel can be estimated using the channel filling concept. Both filling of the channel (*F*) and its value expressed as a percentage (%*F*) were calculated as described by Krasilnikov et al. (1998):

(1) $$\begin{array}{l} F=\left[\left(g_{o}-g_{i}\right)∕g_{i}\right]∕\left[\left(\chi_{o}-\chi_{i}\right)∕\chi_{i}\right] \end{array}$$

where *Go* is the single-channel conductance in a solution without the NE (1 M KCl)*, Gi* is the single-channel conductance in the presence of a solution containing 20% (w/v) NE, and *Xo* and *Xi* are the conductivity of the salt solution without the NE and with 20% (w/v) NE, respectively.

Assuming that the filling of the channel by the two smallest NEs (in our study, ethylene glycol and glycerol) is close to the maximum possible level, then filling can be calculated as a percentage (F%):

(2) $$\begin{array}{l} F\%=\left[\left(2*F_{i}\right)∕\left(F_{1}+F_{2}\right)\right]*100 \end{array}$$

where *Fi* is the filling in the presence of a given NE and *F*1 and *F*2 are the filling in the presence of ethylene glycol and glycerol in the bathing solution, respectively.

The radius of the constriction zone should be equal to the radius of the smallest NE that does not pass freely through the channel and therefore does not fill it by 100%.

### Single-channel conductance at the single-unit level in solvent-free planar lipid bilayers

The ability of the channels formed by the supernatants to transport carcinogenic molecules was evaluated in experiments using planar lipid bilayers, formed according to the monolayer technique of Montal and Mueller (1972). The bilayers consisted of two monolayers juxtaposed and extended across an aperture 50-100 μm in diameter and developing in a 25-μm thick polytetrafluoroethylene (PTFE) film. A hydrophobic film was spread across the aperture by painting the latter with 1 μL of a 1% solution of n-hexadecane in n-hexane. After the hydrophobic film had dried, both chambers were filled with buffer (1 M KCl and 20 mM MES, pH 6). A lipid membrane with an area of ~0.008 mm^2^ was formed by adding 1 μL of a 5 mg/mL solution of DphPC in a solvent mixture of n-pentane to the aperture. Once a single bilayer insertion was obtained, solubilized smoke, prepared as described above, was added and the resulting signal was recorded.

Ag/AgCl electrodes were used to detect the ionic currents, with the live electrode connected to the headstage of an Axopatch 200B amplifier (Axon Instruments) in the voltage clamp mode. Signals were filtered by an on-board low-pass Bessel filter at 10 kHz and recorded onto a computer hard drive with a sampling frequency of 50 kHz. The conductance of the channels was analyzed using Clampfit (Axon Instruments) and Origin (Microcal Software).

## Results

### Bacteria in cigarettes

All cigarettes analyzed contained a significant number of bacteria: between 50 and 1700 colony forming units (CFU)/cm cigarette or the equivalent amount of rolling tobacco. The morphology of all colonies was typical of spore-forming gram-positive bacilli and all of the isolates were confirmed to be spore-forming Gram-positive rods. Specifically, based on their staining, microscopy, and biochemical features, the bacteria could be assigned to *Bacillus* sp., which was confirmed by their 16S rRNA sequence homologies and by MALDI-TOF.

Analyses of the 37 different isolates also showed that all were aerobes or facultative anaerobes, spore-forming, Gram-positive, catalase-positive, and indol-, citrate-, and phenylalanine-negative. Testing for hemolysis, urease, starch hydrolysis, Voges-Proskauer, anaerobic growth, growth on mannitol, oxidation/fermentation, and growth on 6.5% NaCl yielded variable results depending on the isolate (see Supplementary Material Table 1). However, all isolates were susceptible to all of the tested antimicrobials (amoxicillin-clavulanic acid, chloramphenicol, ciprofloxacin, clindamycin, erythromycin, cotrimoxazole, ampicillin, penicillin, vancomycin, cefotaxime, and gentamicin), which is consistent with their soil origin and with the lack of selective pressure in their recent history.

### Bacteria in smoke

Using the “smoker” device it was also possible to isolate bacteria from the “smoker” Ringer ¼ solution contained in the Kitasato flask. The microbial concentration after “smoking” a single cigarette was as high as 200 CFU/ml. When these isolates were re-identified, *Bacillus* was the predominant if not the only genus present.

### Bacteria in lung cancer biopsies

*Bacillus*-like microorganisms were isolated from seven (23.3%) of the 30 biopsies of malignant lung tumors. All tumors that were positive for *Bacillus* were adenocarcinomas. Five were classified as mixed subtype adenocarcinoma with acinar and solid growth, one as mucinous adenocarcinoma and one as an enteric-type large-cell neuroendocrine carcinoma.

These seven isolates, were gram-positive, spore-forming, and catalase-positive and were subsequently identified as *Bacillus* spp. Four of the six biopsies contained more than one *Bacillus*. The colony morphologies of the *Bacillus* spp. from the tumors were quite similar to those of the tobacco isolates, except in the case of one isolate. All of the isolates were susceptible to the tested antibiotics (representatives of the β-lactam, quinolone, aminoglycosides, macrolide, tetracycline, chloramphenicol families) as expected, since these strains have never been under antibiotic selective pressure.

### Biofilm formation

**Figure 2:**
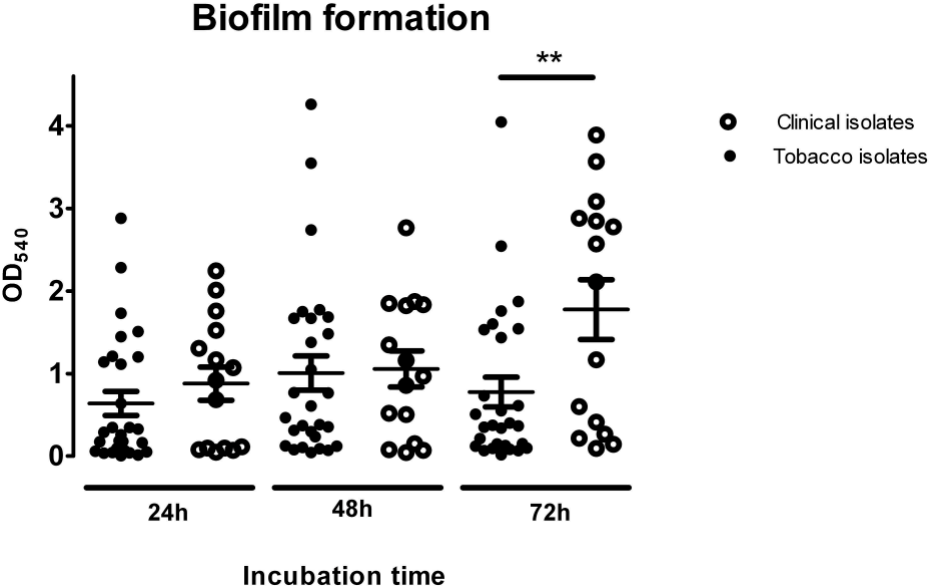
**Biofilm formation analysis of tobacco isolates and clinical strains at different incubation times**. One-way Anova analysis of variance with the Newman-Keuls múltiple-comparison test was used for statistical analysis (^**^P < 0.01).

The ability to form biofilm is regarded not only as a main virulence factor in infectology, but has also been related with one of the reasons why bacteria may induce malignancies. Thus, bacterial biofilm has now been implicated in chronic laryngitis. Among head and neck cancer patients, biofilm is the main reason for the short life cycle of indwelling devices such as voice prostheses and tracheal tubes. Recently, bacterial biofilm has been related to dysplasia and malignancies both as an etiological factor and as a source of complications (Kinnari, 2015). Moreover, it has been pointed out that colonic mucosal biofilms alter the cancer metabolome producing regulators of cellular proliferation and colon cancer growth and potentially affecting cancer development and progression (Johnson et al., 2015). Thus, all of the tobacco- and tumor-derived isolates were tested for their ability to form biofilms (Figure 2). Strong adherence was determined in only 20% of the isolates from tobacco but in 61 % of the isolates from the biopsies (Supplementary Material Table 2).

As a general trend, biofilm biomass increased as a function of time. At 72 h, a peak was reached that was twice as high as the initial reading at 24 h. Among the tobacco isolates, moderately adherent strains accounted for 40% of the total bacteria at 72 h, with the remaining 60% made up of non-adherent, weakly adherent, and strongly adherent bacteria. Among the clinical isolates, strongly adherent isolates predominated, accounting for 60% of the total bacteria, with non-adherent, weakly adherent and moderately adherent strains comprising the remaining 40%.

Bacterial viability after different incubation times was estimated using the resazurin metabolism assay, which showed that almost all of the cells were metabolically actives after 72 h of biofilm incubation.

### Channel formation

**Figure 3:**
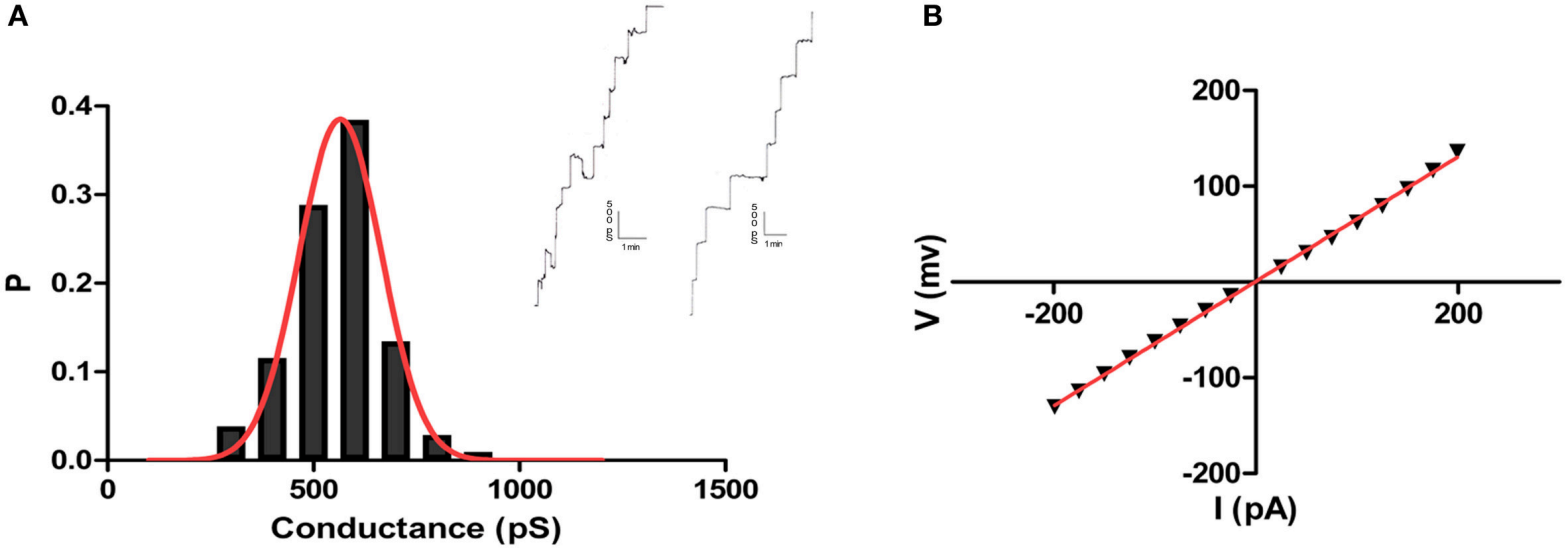
**Pore forming activity of *Bacillus* CP2**. Shown are independent records of single channel insertions in a newly formed DPhPC/n-decane membrane in 1 M KCl and +50 mV applied voltage and the corresponding histogram of the pore forming activity of the toxin using the planar lipid bilayer assay **(A)**. Also, IV curve showing linearity of toxin conductance in a single channel experiment **(B)**.

Analysis of the crude culture supernatants in a planar lipid bilayer apparatus showed a stepwise increase in conductance (Figure 3). These steps were not observed in control experiments in which supernatants from control, non-inoculated medium was added. Thus, the bacterial supernatants likely contained a molecule (or molecules) with channel-forming activity. Since all of the supernatants exhibited similar activity, we focused on one, the supernatant of isolate CP2, for further analysis. Using supernatant CP2, more than 100 single-channel events were detected in 1 M KCl; their average conductance was 565 pS (Figure 3). The conductance of single channel was non-voltage-dependent (Supplementary Material Figure 1).

The single-channel conductance in KCl was a linear function of the electrolyte concentration, suggesting that the channel interior did not contain a binding site for the electrolyte. Channel-insertion events were also studied using different ions and solutes (Table 1), including other cations and organic solutes, to estimate pore diameter. Pore size was estimated by measuring the translocation of PEGs of increasing molecular weights (Table 2). Because NEs are uncharged molecules, they avoid the attraction/repulsion forces that develop between ions and charges in the channel interior. In addition, the determination of channel diameter using NEs is not influenced by the conformation of the pores in the case of oligomeric channels. From the experiments with the selected NEs, the estimated pore diameter was approximately 2.88 nm. These calculations include an estimated error of ±0.1 nm caused by the overlap of the molecular masses of the individual NEs, thus influencing their hydrodynamic radii (Krasilnikov et al., 1992; Nablo et al., 2008). According to our estimations of channel filling, no internal constrictions were present inside the channel.

**Table 1:** **Single channel measurements of *Bacillus* CP2 in different KCl concentrations and different electrolyte solutions**.

**Electrolyte**	**Concentration [γ]**	**χ**	**G**
	**M**	**mS/cm**	**pS**
KCl	0.1 [0.77]	13.1	95
	0.3 [0.68]	37.1	186
	1 [0.60]	106.5	565
	3 [0.56]	291.8	1778
LiCl	1 [0.77]	70.3	345
Kac	1 [0.78]	68.6	306

The CP2 conductance (G) in each salt solution was taken from the highest conductance value observed in a Gaussian distribution of single channel conductance. To analyze CP2 conductance, in each case at least 100 channels were reconstituted in DPhPC membranes at +50 mV applied potential. The solutions were used unbuffered with a pH close to 6 unless otherwise indicated. The conductivity (χ) of each salt solution was measured at room temperature with a conductometer (Knick 703). Ion activities (γ) of the salts at 25°C are given in parentheses.

**Table 2:** **Average single channel conductance of CP2 toxin in the presence of different NEs in the bathing solution**.

**NE**	**Mw**	**r**	**G**	**CP2**			
				**G+NE/G-NE**	**X (mS/cm)**	**Filling**	**%**
None			565		103.5		
Ethylene glycol	62	0.26	277	0.490	68.8	2.07	99.62
Glycerol	92	0.308	281	0.497	69.6	2.08	100.38
Arabinose	150	0.34	278	0.492	66.7	1.87	90.20
Sorbitol	182	0.39	293	0.519	68.0	1.78	85.78
PEG 200	200	0.43	182	0.322	55.0	2.39	115.08
PEG 400	400	0.7	145	0.257	50.0	2.71	130.59
PEG 600	600	0.78	189	0.334	54.1	2.18	105.18
PEG 1000	1000	0.94	171	0.303	52.3	2.35	113.54
PEG 2000	2000	1.22	437	0.774	53.1	0.31	14.82
PEG 3350	3350	1.44	501	0.887	55.6	0.15	7.14
PEG 6000	6000	2.5	540	0.956	50.5	0.04	2.13

Average single channel conductances (G) were calculated from at least 100 conductance steps. The aqueous phase contained 1 M KCl and the corresponding nonelectrolyte at a concentration of 20% (w/v). Vm = +50 mV; T = 23 ± 1.5°C; Mw, molecular mass; r, hydrodynamic radius; χ is the conductivity of the aqueous solutions. Mw and r, as well as the channel filling (F) and percentage of channel filling (%F) were taken and calculated as described elsewhere (Krasilnikov et al., 1992, 1998; Bárcena-Uribarri et al., 2013).

Taken together, these results suggested that small molecules (max. 2000 g/mol) are able to access to the channel interior and therefore to reach the cell interior by diffusion. Among the molecules with a size below the molecular weight of the cut-off are benzopyrenes, suggesting a link between the channel-forming activity of the isolated *Bacillus* sp. and carcinogenesis.

### Channel activity in response to tobacco smoke

**Figure 4:**
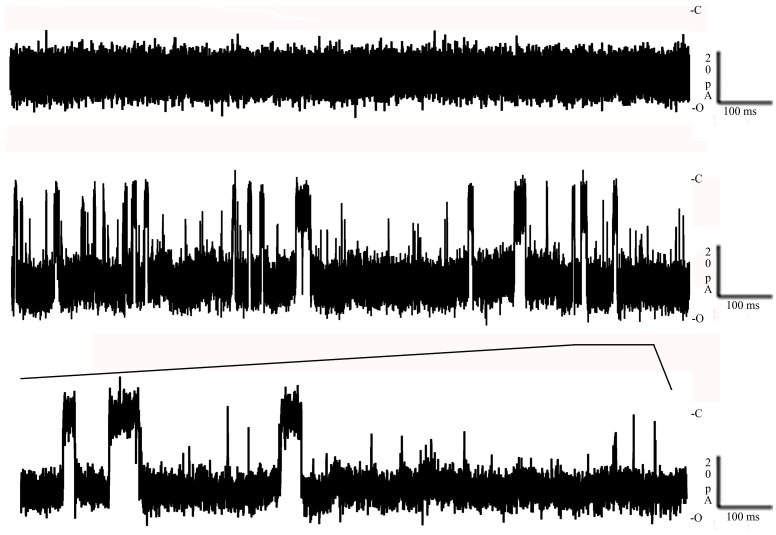
**Toxin interaction with solubilized tobacco smoke**. **Top:** Negative control of the toxin inserted in a newly formed lipid bilayer in the absence of any substrate. **Bottom:** The decrease down to 0 of its conductance in the presence of solubilized tobacco smoke. Both events were recorded at −100 mV at room temperature (C, closed; O, open).

Channel blockage was analyzed to determine whether the membrane channels formed by *Bacillus* products allowed the passage of putative carcinogens present in “smoked Ringer ¼.” In the absence of SR, ion currents through the channels were stable, without fluctuations. Following the addition of SR fluctuations in the ion current indicated strong interactions between the crude material and the channel. As shown in Figure 4, partial and complete blockage of the channel was consistent with the chemical heterogeneity of SR. Thus, its different components seem to be able to interact with the channels formed by the *Bacillus* supernatant, resulting in the internalization of some of these components into the cell. Increasing the SR concentration increased the frequency of the channel-blockage events. Calculation of the residence times of the SR components showed that they were highly heterogeneous, suggesting the involvement of different molecular species. The number of binding events was strongly dependent or independent of the polarity of the applied voltage, with stronger binding events occurring at negative rather than at positive voltages. This mechanism of voltage asymmetry is not completely understood.

## Discussion

This study demonstrated the surprisingly large bacterial burden of cigarettes. These bacteria were almost always spore-forming Gram-positive bacteria identified as *Bacillus* sp. Aerobic spore-forming members of the genus *Bacillus* are commonly isolated from many types of soil at a range of depths and altitudes and under various climatic conditions. Thus, *Bacillus* can also be expected in essentially all habitats where tobacco is cultivated and manipulated. Moreover, their spore-forming ability ensures that they are well-adapted to survive the storage and curing conditions (low humidity, lack of nutrients, etc.) as well subsequent processing steps of tobacco. The ecology of *Bacillus* species in soil is far from complete but their contribution to the soil microbiota and to the rhizosphere of some plants is well established. Moreover, most *Bacillus* produce antibiotics and some cases (e.g., *B. cereus*) secrete toxins, including those that cause food poisoning (Lund and Granum, 1996). *Bacillus* species also include *B. thuringiensis*, the commercially exploited insect pathogen that produces insecticidal proteins (Crickmore, 2006), and *B. anthracis*, the classical pathogen responsible for anthrax (Liu et al., 2014).

According to our results, a smoker consuming 20 cigarettes/day will aspirate between 4000 and 13,600 spores/day in an almost slow and continuous “lung inoculation.” Although *Bacillus* species are mainly associated with gastrointestinal disorders and eye infections, they are also opportunistic pathogens associated with necrotizing infections, endocarditis, periodontitis, osteomyelitis, sepsis, liver abscess, pneumonia, and meningitis, especially in postsurgical patients, immunosuppressed individuals, intravenous-drug abusers, and neonates. Nonetheless, the exact mechanism by which *Bacillaceae* cause these clinical infections is poorly understood (Kamar et al., 2013). Despite lungs have historically been considered sterile in health, the emergence of modern methods for molecular detection have demonstrated that the idea of sterility was due to the failing in detecting cultivable microorganisms. Progress in this field lead to the demonstration that bacterial DNA is almost always present in human lung. Thus, in healthy lung *Bacteroides, Prevotella, Veillonella, Streptococcus, Staphylococcus, Pseudomonas, Haemophilus, Moraxella, Neisseria, Acinetobacter*, and *Escherichia* have been described as the components of human lung microbiome. In cystic fibrosis it becomes easy the detection of bacteria by culture, in this case they are truly pathogens such as *Pseudomonas* or *Staphylococcus* or infrequent ones such as *Stenotrophomonas maltophilia* or *Burkholderia cepacia*. Asthma and COPD patients have a microbiome with increased proteobacteria series (Dickson et al., 2013; Sze et al., 2015). The presence of *Bacillus* in the lung is poorly documented. Only a few reports have been published. In 1976 *Bacillus sphaericus* (*Lysinibacillus sphaericus*) was described in a case report as cause of a lung unusual mucous mass (a pseudotumour; Isaacson et al., 1976) and occasional reports of *Bacillus* in patients with severe pathologies have been published (Logan, 1988).

Our study demonstrated the presence of *Bacillus* spores (and possible vegetative bacteria) in cigarettes. Moreover, the results implied that the spores were resistant to combustion of the cigarette and therefore presumably retained their ability to enter the respiratory tract. Spore inhalation is not significantly hindered by cigarette filters. Pauly et al. (2008) observed tobacco flakes lying loosely on the cut surface of the filters of cigarettes in newly opened packs. They succeeded in growing bacteria from these filters and subsequently predicted that diverse microbes and microbial toxins are carried by the tobacco flakes. They also suggested that particles formed from the flakes are released from the filter during smoking and enter the mainstream smoke, which is inhaled deep into the lung. With our smoker device, the suspension of small flakes coming from the cigarettes could be readily observed. According to our findings, neither the tobacco flakes adhering to the filter nor the bacteria that were “smoked” along with the tobacco was retained by filter, thus allowing the access of both to the respiratory tract. The study of Pauly et al. highlighted the need to explore the effect of inhaling bacteria and their toxins.

In seeking evidence of a direct relationship between the presence of *Bacillus* and the occurrence of lung cancer, we considered that bacterial spores in the lung were inducers of inflammation and therefore favored cancer development. However, tests of the mutagenic activity of the supernatants of the cultured isolates (Ames test) were consistently negative. We then considered the possibility that the isolates secreted biologically active molecules into the medium, as shown for several other spore-forming Gram positive bacilli, such as *B. cereus* and others, which release toxins into the surrounding medium. Membrane injury induced by these toxins can be evaluated electrophysiologically. Our initial hypothesis was that the toxins form transmembrane channels that serve as an open door allowing carcinogenic products to reach molecular targets inside the cell. Thus far, while the SR material was clearly shown to contain channel-forming activity, our attempts to purify the molecule responsible for channel formation, including gel electrophoresis, chromatography, and chemical fractionation, have been unsuccessful. One very plausible reason is that the channel consists of two or more components, as is the case in the toxins formed by other *Bacillaceae* (Miles et al., 2002; Nishiwaki et al., 2007; Fagerlund et al., 2008; Nablo et al., 2008; Zhao et al., 2013). Work is currently in progress to purify to homogeneity the channel-forming molecule(s).

## Funding

AM was recipient of a predoctoral fellowship (APIF). This work was partially funded by the program “Ajuts a la Recerca del Campus de Bellvitge” (ACESB 0098) and a mobility fellow from the International Health Sciences Campus of Excellence of the University of Barcelona (HUBc).

### Conflict of interest statement

The authors declare that the research was conducted in the absence of any commercial or financial relationships that could be construed as a potential conflict of interest.
