# Supplementary material for: Toxins Secreted by Bacillus Isolated from Lung Adenocarcinomas Favor the Penetration of Toxic Substances
Source: Front Microbiol. 2015 Nov 23;6:1301. doi: 10.3389/fmicb.2015.01301 (PMC4655230; doi:10.3389/fmicb.2015.01301)
Supplement: Supplementary file 1 [file DataSheet1.DOCX]

**Supplementary materials**

**Table 1.** Bacterial biochemical characterization.

| **Isolate** | **Haem** | **Cat** | **Spo** | **OF** | **Cit** | **VP** | **Starch** | **Indol** | **Phen** | **Urease** | **6,5% NaCl** | **Man** |
| --- | --- | --- | --- | --- | --- | --- | --- | --- | --- | --- | --- | --- |
| A11 | γ | + | + | OF | - | + | - | - | - | - | + | - |
| A12 | γ | + | + | OF | - | - | - | - | - | - | + | - |
| A13 | γ | + | + | OF | - | - | - | - | - | - | + | - |
| A14 | γ | + | + | F | - | + | + | - | - | - | + | - |
| A15 | γ | + | + | O | - | - | + | - | - | - | + | + |
| CL11 | γ | + | + | OF | - | + | - | - | - | - | + | - |
| CL13 | β | + | + | O | - | + | + | - | - | + | + | - |
| DN11 | β | + | + | O | - | - | - | - | - | - | + | - |
| DN12 | β | + | + | OF | - | + | + | - | - | * | + | - |
| DN13 | β | + | + | F | - | + | - | - | - | - | + | - |
| DN14 | β | + | + | O | - | + | - | - | - | - | + | - |
| DN15 | β | + | + | O | - | + | - | - | - | - | + | - |
| DR11 | γ | + | + | OF | - | + | + | - | - | + | + | + |
| DR12 | γ | + | + | OF | - | + | + | - | - | - | + | - |
| DR13 | γ | + | + | F | - | + | + | - | - | - | + | - |
| DR14 | γ | + | + | OF | - | + | - | - | - | - | + | + |
| DR15 | γ | + | + | OF | - | - | + | - | - | - | + | + |
| DR16 | γ | + | + | F | - | + | + | - | - | + | + | + |
| M11 | β | + | + | OF | - | + | + | - | - | + | + | - |
| M12 | β | + | + | OF | - | - | + | - | - | - | + | - |
| M13 | β | + | + | OF | - | + | - | - | - | - | + | - |
| M14 | β | + | + | OF | - | - | - | - | - | - | + | - |
| M15 | β | + | + | F | - | + | + | - | - | - | + | - |
| M16 | β | + | + | OF | - | + | + | - | - | - | + | - |
| M17 | γ | + | + | OF | - | + | - | - | - | - | + | - |
| Po11 | γ | + | + | OF | - | + | - | - | - | - | + | - |
| Po12 | γ | + | + | OF | - | + | - | - | - | - | + | - |
| Po13 | γ | + | + | F | - | + | + | - | - | - | + | - |
| Po14 | γ | + | + | OF | - | + | - | - | - | - | + | - |
| P11 | γ | + | + | O | - | + | - | - | - | - | + | + |
| P12 | γ | + | + | O | - | + | + | - | - | - | + | + |
| P13 | γ | + | + | O | - | - | + | - | - | - | + | + |
| P14 | γ | + | + | OF | - | + | + | - | - | + | + | + |
| P15 | β | + | + | F | - | - | + | - | - | - | + | + |
| P16 | γ | + | + | O | - | - | + | - | - | - | + | - |

Haem: haemolysis; cat: catalase; spore: sporulation; cit: citrate; phen: phenylalanine; man: mannitol

**Figure 1.** **Voltage dependence of the *Bacillus* CP2 toxin.** Shown are the average values recordings of single channel insertions in a newly formed DPhPC/n-decane membrane with over 100 insertions in 1 M KCl at different applied voltages.

**Table 2**. Biofilm formation ability. CV Cristal Violet, AB Resazurin at different times of incubation.

CV legend. white: non-adherent; purple: weakly adherent; green: moderately adherent; red: strongly adherent.

| **Isolate** | **CV 24h** | **CV 48h** | **CV 72** | **AB 24h** | **AB 48h** | **AB 72h** |
| --- | --- | --- | --- | --- | --- | --- |
| DN11 | 0,015 | 0,354 | 0,067 | 40984 | 38746 | 31641 |
| DN12 | 0,095 | 0,123 | 0,148 | 42984 | 39843 | 36527 |
| DN13 | 0,259 | 0,298 | 0,365 | 36784 | 39587 | 32946 |
| CL11 | 0,345 | 0,467 | 0,507 | 45988 | 40123 | 39516 |
| CL13 | 0,289 | 0,311 | 0,339 | 32567 | 38756 | 30297 |
| DR12 | 0,036 | 0,091 | 0,121 | 3121 | 13086 | 19434 |
| DR13 | 0,163 | 0,380 | 0,399 | 38185 | 41221 | 42787 |
| DR14 | 0,044 | 0,079 | 0,092 | 29275 | 10718 | 8932 |
| DR15 | 0,051 | 0,071 | 0,082 | 3114 | 6772 | 7547 |
| DR16 | 1,113 | 1,379 | 1,435 | 61476 | 50823 | 49854 |
| Po12 | 0,060 | 0,122 | 0,152 | -196 | 13202 | 15898 |
| Po13 | 1,201 | 1,481 | 1,543 | 61476 | 49830 | 41345 |
| Po14 | 1,508 | 1,668 | 1,601 | 61367 | 46058 | 39346 |
| P11 | 0,184 | 0,106 | 0,098 | 4424 | 8780 | 10121 |
| P12 | 1,211 | 1,671 | 1,871 | 61476 | 52090 | 49811 |
| P13 | 1,729 | 2,738 | 2,543 | 60767 | 46272 | 40856 |
| P15 | 0,347 | 0,606 | 0,551 | 55026 | 40565 | 35676 |
| P16 | 0,329 | 0,235 | 0,211 | 61336 | 48917 | 42896 |
| A12 | 0,007 | 0,04375591 | 0,016 | 28033 | 43701 | 19752 |
| A13 | 0,040 | 0,765 | 0,130 | 25539 | 11409 | 21160 |
| A14 | 1,140 | 1,684 | 0,352 | 56267 | 47551 | 30930 |
| A15 | 0,179 | 0,770 | 0,730 | 15807 | 30442 | 11379 |
| A16 | 0,173 | 0,369 | 0,613 | 19793 | 23086 | 6845 |
| P14 | 2,881 | 4,260 | 4,046 | 58013 | 50344 | 48192 |
| M13 | 0,638 | 1,048 | 0,373 | 29759 | 41925 | 23340 |
| M14 | 0,063 | 1,771 | 0,067 | 30360 | 34947 | 17067 |
| M16 | 2,284 | 3,549 | 1,530 | 56496 | 47081 | 48131 |
| M17 | 1,446 | 1,751 | 1,758 | 51353 | 44812 | 28926 |
| T.VR.1 | 0,917 | 1,821 | 2,57 | 47525 | 26112 | 45609 |
| T.VR.3 | 1,758 | 1,833 | 3,082 | 48339 | 42957 | 37363 |
| T.VR.5 | 0,096 | 0,045 | 0,145 | 21137 | 4990 | 16882 |
| T.VR.6 | 0,047 | 0,080 | 0,217 | 11833 | 15052 | 14085 |
| CP1 | 1,524 | 1,851 | 3,889 | 45527 | 46773 | 40442 |
| CP2 | 1,072 | 1,347 | 2,879 | 38041 | 48292 | 46780 |
| CP3 | 0,686 | 1,161 | 2,845 | 36701 | 46658 | 42875 |
| CP4 | 2,244 | 1,874 | 3,567 | 49994 | 51337 | 48956 |
| P.VR.1 | 1,304 | 0,521 | 2,779 | 41176 | 17713 | 32413 |
| P.VR.3 | 0,072 | 0,503 | 0,600 | 13114 | 4565 | 28133 |
| P.VR.4 | 0,114 | 0,151 | 0,411 | 17804 | 20205 | 17634 |
| P.VR.5 | 2,010 | 2,764 | 2,111 | 46154 | 38359 | 44155 |
| 1T | 0,098 | 0,967 | 1,168 | 13481 | 23556 | 24485 |
| 8P | 1,169 | 0,857 | 0,268 | 46473 | 23977 | 19960 |
